# Supplementary material for: Effect of Osimertinib on CTCs and ctDNA in EGFR Mutant Non-Small Cell Lung Cancer Patients: The Prognostic Relevance of Liquid Biopsy
Source: Cancers (Basel). 2022 Mar 19;14(6):1574. doi: 10.3390/cancers14061574 (PMC8946716; doi:10.3390/cancers14061574)

Supplementary Materials and Methods

Eligibility criteria: Patients have to fulfill the following inclusion and exclusion criteria: age >18 years, stage IIIB not amenable to radical therapy or stage IV, Performance Status (ECOG) of 0-1, measurable or evaluable disease, adequate organ function tests (Hb≥10g/dL, WBC≥3.0 x 10^9/L , neutrophils count≥1.5 x 10^9/L, platelets≥100 x 10^9/L, Creatinine clearance≥50 mL/min, Total bilirubin≤1.5 X UNL, AST, ALT and ALP≤ 2.5 x UNL), a life expectancy of at least 3 months and normal QT interval in ECG. CNS metastases were allowed provided that they had been irradiated and the patient was clinically stable. Patients with a history of serious drug allergy, clinically important abnormalities of cardiac rhythm, severe or uncontrolled systemic liver disease, including those with known hepatitis B, hepatitis C, and Human Immunodeficiency virus (HIV) infection, interstitial lung disease or pulmonary fibrosis, pregnancy and lactation, as well as other concurrent active malignancy were excluded from the study.

Immunofluorescence staining: For CK staining, two different antibodies were used as a cocktail, namely A45-B/B3 anti-mouse Ab recognizing CKs 8/18/19 (Micromet Munich, Germany) and an anti-mouse Ab against CK7 (Abcam, Cambridge, UK), since CK7 is highly expressed in NSCLC tumor cells (37). Alexa 488 (Invitrogen Carlsbad, CA, USA) anti-mouse was used as a secondary antibody. Spots were then incubated with anti-CD45 Ab conjugated with Alexa 647 (Novus Biologicals, USA). Consequently, slides were stained with an anti-rabbit antibody against VIM (Abcam, Cambridge, UK) and Alexa 555 (Novus Biologicals).

The NSCLC cell lines H460 (large cell lung cancer), H1299 (lung, derived from metastatic lymph node), HCC827 (adenocarcinoma lung cancer) and SK-MES-1 (squamous cell carcinoma), obtained from the American Type Culture Collection (ATCC; USA), were used as positive controls for the IF experiments while negative controls were prepared by omitting the corresponding primary antibodies and incubating the cells with the matching IgG isotypes bound to the corresponding fluorochromes.

PNA-Q-PCR assay: Amplification was performed in a final volume of 12.5 μL, using 3 μL (~4.5 ng) for exon 21 analysis or 1 μL (~1.5 ng) for exon 19, p.T790M, p.C797S, of ctDNA; 6.25 μL of Genotyping Master Mix (Applied Biosystems, USA); 0.96 pmol of each primer; 1.2 pmol of probes and 6.25 pmol (for

exon 21, p. T790M, and p.C797S) of PNA. Samples were submitted to 50 cycles of 15 sec at 92°C and 1.5 min at 60°C, in a QuantStudioTM 6 real time PCR System (Applied Biosystems/Thermo Fisher Scientific, MA, USA). The sequence of the primers, probes and PNAs used have been described elsewhere (38, 39). Analyses were carried out in duplicates and all samples were also assayed in the absence of PNA to confirm the presence of ctDNA. Genomic DNAs from cell lines at 5ng/μL were used as positive and negative controls. Extraction and non-template controls were added in each run. A sample was considered positive if the same mutant allele amplified in the two duplicates in the presence of PNA as previously described (38, 39). For detection of *EGFR* exon 18 mutations (p.G719X) and exon 20 insertions (not included in the PNA-Q-PCR assay) Therascreen® EGFR RGQ PCR Kit V1 (Qiagen) was used, following the manufacturer's instructions. The assay also allowed estimation of the relative abundances of mutant alleles in positive samples.

Supplementary Figure S1. Consort Diagram

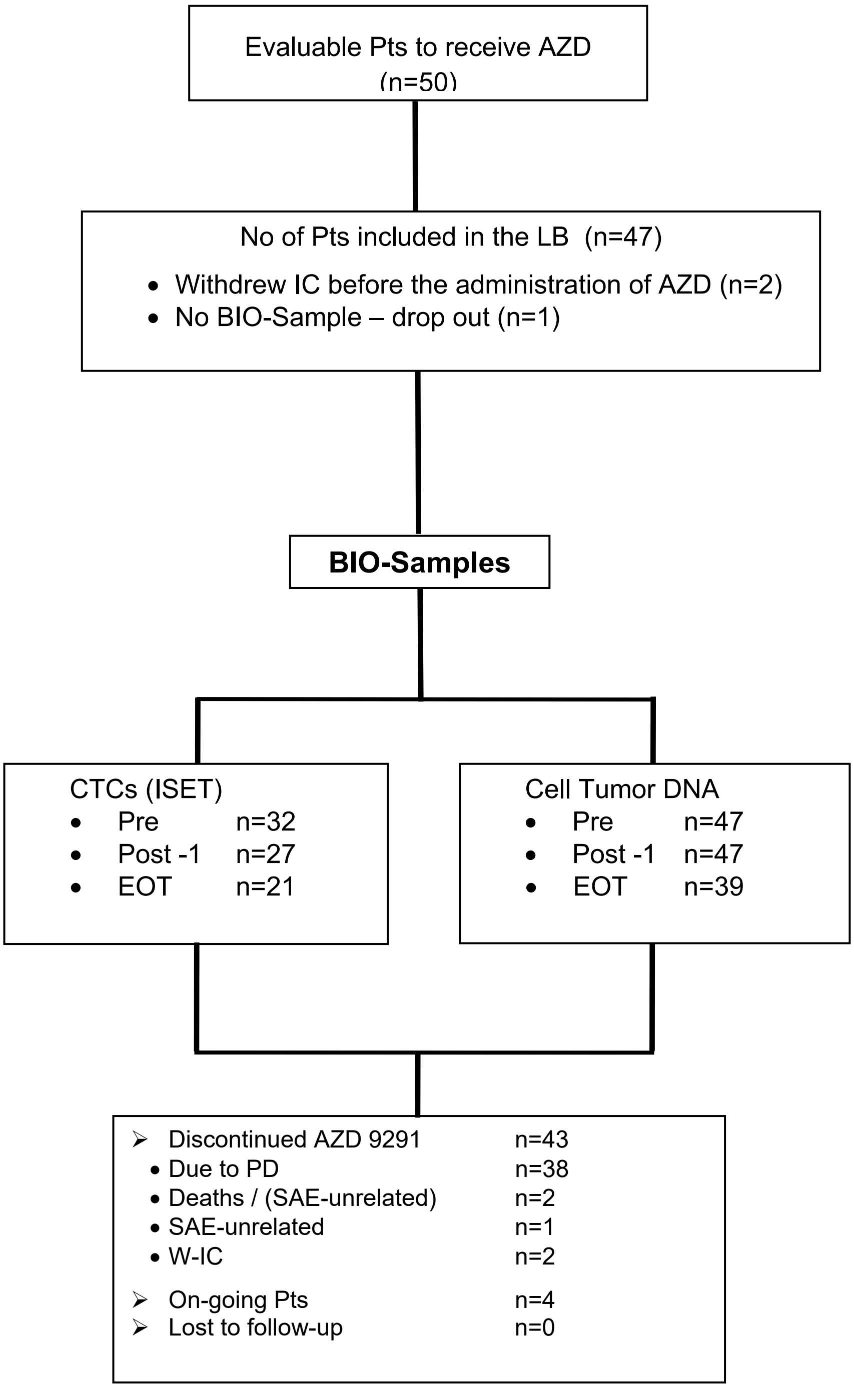

Supplementary Table S1. Somatic EGFR mutations

Exon	Mutation	Patients N (%)
**18**	G719X+Exon 20 (S768I)	3 (6.4%)
**19**	del19	16 (30.0%)
	del19+Exon 20 (T790M)	11 (23.4%)
	del19+Exon 20 (H773_V774)	1 (2.1%)
**20**	H773_V774insNPH	1 (2.1%)
**21**	L858R	11 (23.4%)
	L858R+Exon 20 (T790M)	3 (6.4%)
	L861Q	1 (2.1%)
		Total N= 47

Supplementary Table S2. Adverse Events possibly or probably related to study treatment

	**GrI**		**GrII**		**GrIII**		**GrIV**	
	**N**	**%**	**N**	**%**	**N**	**%**	**N**	**%**
Lympopenia	-	-	2	4.3				
Leukopenia	-	-	-	-	-	-	1	2.1
Neutropenia	1	2.1	-	-	1	2.1	-	-
Febrile neutropenia	-	-	-	-	1	2.1	-	-
Anemia	3	6.4	-	-	1	2.1	-	-
Thrombocytopenia	2	4.3	-	-			1	2.1
Nausea	3	6.4	1	2.1	-	-	-	-
Vomiting	1	2.1	1	2.1	-	-	-	-
Diarrhea	6	12.8	-	-	-	-	-	-
Constipation	3	6.4	-	-	-	-	-	-
Skin toxicity	5	10.6	-	-	-	-	-	-
Allergy	4	8.5	-	-	-	-	-	-
Fatigue	4	8.5	2	4.3	1	2.1	-	-
Anorexia	4	8.5	2	4.3	-	-	-	-
Edema	1	2.1	-	-	-	-	-	-
Nail disorders	4	8.5	-	-	-	-	-	-

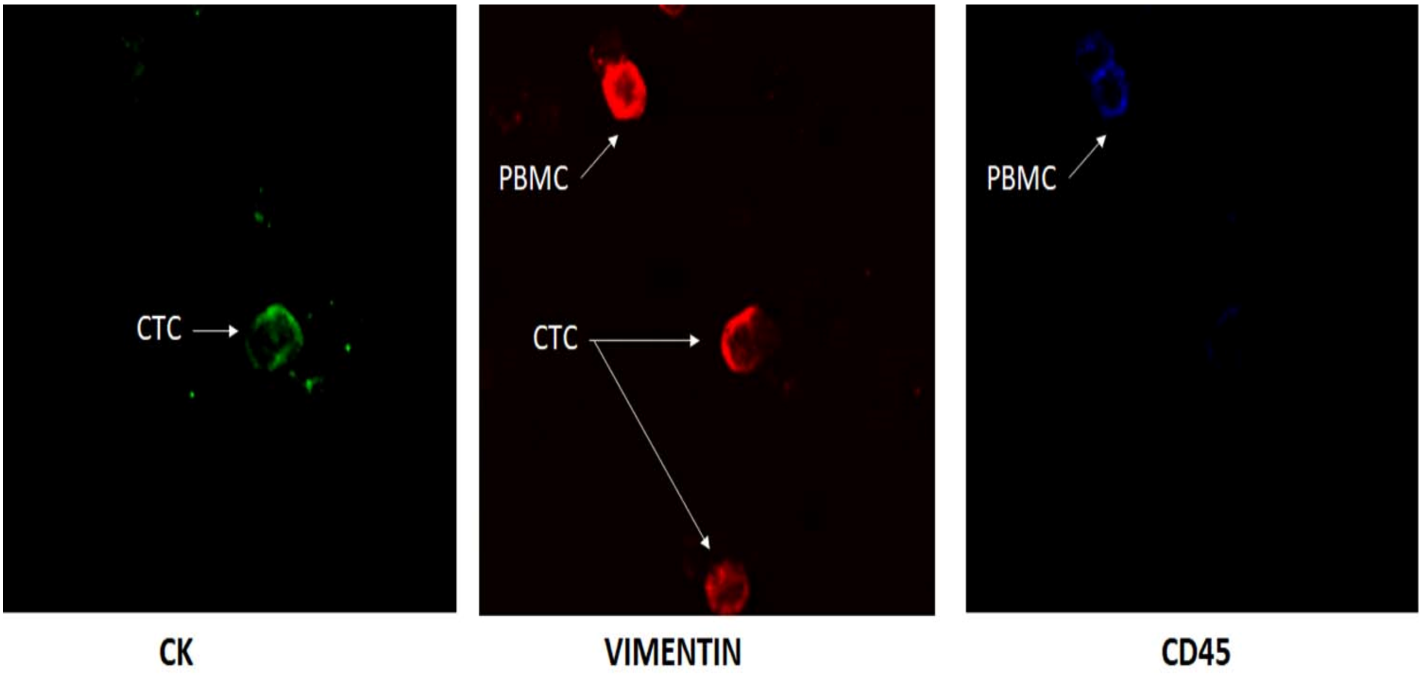

Supplementary Figure S2. Expression of Cytokeratin (CK) and Vimentin (VIM) in patients' CTCs.

Representative confocal laser scanning micrographs of patients' CTCs (x40) stained for CK (green), VIM (red), CD45 (blue) and Dapi (not shown because our confocal has no laser for Dapi)

Supplementary Table S3. Changing status in CTCs and ctDNA

	Baseline / Post-1	
	**CTCs (n=23)**	**ctDNA (n=47)**
	**N (%)**	**N (%)**
**Pre (+) / Post-1 (+)**	8 (34.8)	12 (25.5)
**Pre (+) / Post-1 (-)**	8 (34.8)	17 (36.2)
**Pre (-) / Post-1 (+)**	3 (13.0)	1 (2.1)
**Pre (-) / Post-1 (-)**	4 (17.4)	17 (36.2)
	**Post-1 / EOT**	
	**CTCs (n=15)**	**ctDNA (n=39)**
	**N (%)**	**N (%)**
**Post-1 (+) / EOT (+)**	6 (40.0)	12 (30.8)
**Post-1 (+) / EOT (-)**	1 (6.7)	-
**Post-1 (-) / EOT (+)**	5 (33.3)	12 (30.8)
**Post-1 (-) / EOT (-)**	3 (20.0)	15 (38.5)

Supplementary Table S4. Liquid - Changing status [ctDNA and/or CTCs (ISET) positive]

	Baseline / Post-1 (n=47)
	**N (%)**
**Pre (+) / Post (+)**	20 (42.6)
**Pre (+) / Post (-)**	18 (38.3)
**Pre (-) / Post (+)**	3 (6.4)
**Pre (-) / Post (-)**	6 (12.8)
	**Post-1 / EOT (n=39)**
	**N (%)**
**Pre (+) / Post (+)**	18 (46.2)
**Pre (+) / Post (-)**	3 (7.7)
**Pre (-) / Post (+)**	11 (28.2)
**Pre (-) / Post (-)**	7 (17.9

Supplementary Figure S3. PFS for Patients with detectable and non-detectable LB at baseline

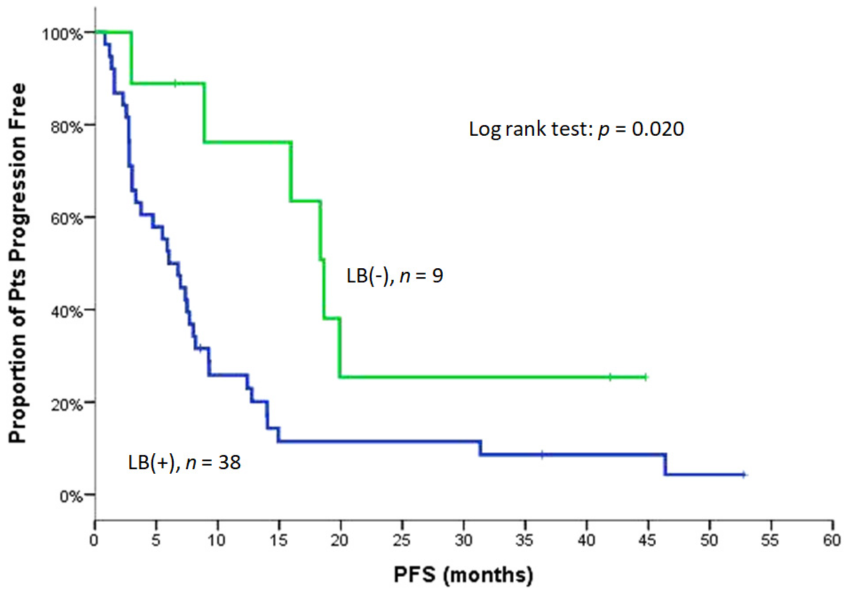

Supplement: Supplementary file 1 [file cancers-14-01574-s001.zip › cancers-1601053-supplementary.pdf]
